# Macrophage Activation Syndrome/Secondary Hemophagocytic Lymphohistiocytosis in Adult‐Onset Still's Disease: An Uncommon Initial Presentation in a Young Nepalese Female: A Case Report

**DOI:** 10.1002/ccr3.70128

**Published:** 2025-01-12

**Authors:** Kanchan Bishwakarma, Kajal Bishwakarma, Sunil Bogati, Saket Jha

**Affiliations:** ^1^ College of Medicine Nepalese Army Institute of Health Sciences Sanobharyang Kathmandu Nepal; ^2^ Institute of Medicine Tribhuvan University Maharajgunj Kathmandu Nepal; ^3^ NYMC–St. Mary's General Hospital and St. Clare's Denville Hospital Denville New Jersey USA; ^4^ Department of Rheumatology, Institute of Medicine Tribhuvan University Maharajgunj Kathmandu Nepal

**Keywords:** adult onset stills disease, ferritin, hemophagocytic lymphohistiocytosis, lactate dehydrogenase, neutrophilic leukocytosis

## Abstract

Hemophagocytic lymphohistiocytosis (HLH), is a fatal systemic hyperinflammatory syndrome. HLH may be due to immunosuppression, infections, cancer, or autoimmune diseases with fever and cytopenia. HLH which occurs in adult‐onset Stills disease (AOSD) is called secondary HLH, also known as macrophage activation syndrome (MAS). Here, we present a case of a 36‐year‐old Nepalese female, with no known comorbidities presented with a history of fever, sore throat, multiple joint pain, fluctuating rash, hair loss, and unintentional weight loss for a month. She was hypotensive, with a high‐grade fever. She had swollen eyelids, and erythematous macular rashes in the face, trunk, and extremities with the rest of the systemic examinations normal. Investigation showed leukocytosis, with anemia, and a blood smear showed neutrophilic leukocytosis. ESR/CRP and lactate dehydrogenase (LDH) were elevated, and ferritin was 38,291 ng/mL. Tropical disease screening, blood culture, viral serologies, imaging for malignancies, and autoimmune disease panels were negative. She met the diagnostic criteria for AOSD. MAS was suspected of abnormally high ferritin levels, and a bone marrow aspiration biopsy was done. She was given IV steroids with some improvement. The biopsy showed hypercellular marrow with erythroid hyperplasia, dyserythropoietic changes, and increased macrophages with phagocytic activity suggestive of MAS. She was started on dexamethasone and cyclosporine which eventually improved her condition. Several complications can arise in AOSD, around 15% of these patients can have MAS which is regarded as one of the most severe complications. With studies showing a mortality rate of more than 50% in patients of AOSD with MAS which is five times more than the mortality rate with AOSD alone, understanding this combined picture and timely aggressive treatment has a huge importance.

**Summary**

- MAS is an underdiagnosed life‐threatening complication of adult‐onset Stills disease (AOSD).
- The flare of leukopenia or thrombocytopenia and extremely high serum ferritin levels in AOSD suggest macrophage activation syndrome (MAS).
- As hemophagocytosis on bone marrow biopsy is not mandatory for its diagnosis, first‐line treatment with high‐dose steroids must be timely started for such patients.

## 1 Introduction

Hemophagocytic lymphohistiocytosis (HLH) is a fatal systemic hyperinflammatory syndrome occurring due to immunosuppression, infections, cancer, or autoimmune diseases with fever and cytopenia being typical manifestations [1]. The diagnosis of HLH is based on the following criteria: fever; splenomegaly; cytopenia, elevated serum ferritin, reduced fibrinogen and elevated fasting triglyceride levels, elevated CD25 level, reduced natural killer (NK) cell activity or hemophagocytosis in the bone marrow and lymph nodes [1, 2]. An autoimmune condition known as adult‐onset Still's disease (AOSD) which mostly affects joints in adults may put a patient at risk for secondary HLH, also known as macrophage activation syndrome (MAS). MAS in an AOSD patient can be found in the people of European and African countries. This case is an uncommon case from Nepal, of MAS as the initial presentation of an AOSD.

## 2 Case Presentation

### 2.1 Case History/Examination

A 36‐year‐old Nepalese female, from the hilly region with no known prior comorbidities presented to the ER of a tertiary care center with complaints of fever, joint pain, and rash for a month. Fever was insidious in onset, continuous, maximum recorded to be 104.5 F, associated with chills and rigors, associated with generalized body aches, unrelieved by paracetamol. She had multiple joint pain, involving more than five joints associated with swelling, warmth, and stiffening which felt prominent during episodes of fever. She explained she had pink‐colored rashes that began from bilateral upper and lower extremities sparing palms and soles spreading to the abdomen, back, and face which was associated with itching and got prominent during episodes of fever. She also gave a history of weight loss of around 6 kg in a month, loss of appetite, constipation, fatigue, sore throat, painful lesions in the oral cavity, hair loss, and dry eyes. She denied headache, photophobia, neck stiffness, altered sensorium, loss of consciousness, discharge from eyes and ears, cough, chest pain, hemoptysis, shortness of breath, palpitations, vomiting, pain abdomen, and bowel/bladder issues. She had a history of multiple spontaneous pregnancy losses in the past. She had no history of similar illness in her family. On examination, she was ill‐looking, tachycardic, hypotensive, febrile with a temperature of 104°F, tachypneic, and saturating at 90% on room air. There was swelling of bilateral eyelids, and erythematous macular rashes in the face, trunk, and extremities.

## 3 Methods (Differential Diagnosis, Investigations, and Treatment)

Infectious diseases (tropical and viral), autoimmune, and rheumatological conditions (systemic lupus erythematosus and rheumatoid arthritis), AOSD, MAS, and malignancy (lymphoma or leukemia) are important differential diagnoses for the patient's symptoms. In this case, a systematic approach was used to rule out differential diagnosis for the patient's clinical presentation. Initial investigations revealed hemoglobin 12.1 g%, total leukocyte count (TLC) 24,400/mm^3^, with differentials of neutrophils 75%, lymphocytes 11%, monocyte 10%, eosinophils 3%, basophil 1%, and the absolute platelet count was 209,000/mm^3^. ESR came to 58 mm/h, C‐reactive protein was 101.66 mg/L, and lactate dehydrogenase (LDH) was 505 U/L. She was assessed to have septic shock and managed with fluids, pressors, doxycycline, and meropenem along with acetaminophen. To rule out tropical and viral infections, a tropical panel and viral serologies were sent. Test for malarial parasites, rk39, scrub typhus IgM/IgG, Brucella Ab came negative. Blood culture and urine culture were negative. Viral serology for HIV, hepatitis B/C, and EBV were negative. To further investigate autoimmune and rheumatologic conditions, RA factor, and antinuclear antibodies (ANA) tests were sent, both of which returned negative. A CT scan of the chest and abdomen was performed to rule out malignancies. The imaging findings revealed mild hepatomegaly, bilateral renal cortical cysts classified as Bosniak category I, and mild bilateral pleural effusion. Furthermore, peripheral blood smear showed persistent neutrophilic leukocytosis. With the joint involving febrile condition and negative septic workup, she was diagnosed with AOSD. She had a persistent rash, high‐grade fever, joint pain, myalgia, and shortness of breath. She was bradycardic and normotensive with hypoxemia. Ferritin level was 38,291 ng/mL causing suspicion of MAS in AOSD. Injection of methylprednisolone 500 mg was started for 3 days. Bone marrow aspiration and biopsy were sent which showed hypercellular marrow with erythroid hyperplasia with hemophagocytic activity suggestive of MAS in AOSD. ESR was 15 mm/h, fibrinogen 106 mg/dL, TLC 6200/mm^3^, Hb 11.3 g%, platelet count 123,000/mm^3^ suggestive of slow progression toward pancytopenia. IL 6 was 84.74 pg/mL, CD 16.56 was 4.5%, absolute CD 16^+^56 was 57/μL, CD25 (IL 2 receptor) was 00%. The renal function test was normal. The liver function test was normal. Her symptoms improved initially with IV steroids, but while on oral prednisolone, she started deteriorating with high fever, pancytopenia, and low fibrinogen. She got started on IV dexamethasone 10 mg once daily and tablet cyclosporine 100 mg twice daily which eventually improved her condition. She had vesicular lesions over her genitals shown to be likely toxic epidermal necrosis involving pilosebaceous structures. She was given acyclovir, etoposide, and IV dexamethasone, alternate‐day cotrimoxazole for the genital lesions.

## 4 Results

Following the treatment, her fever eventually subsided, her TLC, hemoglobin, and platelet count normalized and her vitals stabilized.

## 5 Discussion

AOSD is an inflammatory joint condition occurring in individuals > 16 years old, with two peak onset age groups (15–25 and 36–46 years) and a higher prevalence in females [2]. Diagnosis, according to Yamaguchi criteria, requires five features, with at least two being major criteria. The major criteria include a 1‐week spiking fever, a transient salmon‐pink rash, arthritis lasting 2 weeks or more, and leukocytosis with mainly neutrophils. Minor criteria involve sore throat, lymphadenopathy, hepatomegaly or splenomegaly, abnormal liver function studies, and negative ANA and RF tests. AOSD shares similarities with systemic juvenile idiopathic arthritis (SJIA) but differs in the age of onset (AOSD > 16 years, SJIA < 16 years). The exact pathway is unknown, but AOSD patients exhibit reduced NK cell and cytotoxic T cell function, along with decreased expression of perforin and granzyme proteins mediating cytotoxic activity [3]. Our patient met the criteria to diagnose her as AOSD.

AOSD has been associated with markedly elevated serum ferritin concentrations, which can be seen in more than 90% of patients. Hepatocytes increase ferritin synthesis as a part of the acute phase response to inflammatory cytokines and extremely high levels of ferritin seen in AOSD can signal a dysregulated feedback loop that increases autoinflammatory response. High levels (> 3000 ng/mL, some > 10,000 ng/mL) signal a dysregulated feedback loop, correlating with disease activity. The degree of ferritin elevation correlates with AOSD disease activity and has been suggested as a serological marker to monitor the response to treatment. Levels rise further with AOSD complicated by MAS/Secondary HLH, a fatal hyperinflammatory syndrome. HLH may occur from immunosuppression, infections, cancer, or autoimmune diseases [1]. Our patient had a fever, cytopenia, elevated serum ferritin, and hemophagocytosis in bone marrow meeting the criteria for MAS/Secondary HLH.

She had symptoms like fever, chills, hypotension, tachypnea, and marked leukocytosis which could be speculated to be from some infection. Viral panels namely viral hepatitis and EBV came negative. The absence of features of infections suggested non‐infectious pathology in our patient. She had not consumed any medications known to cause adverse reactions which made drug reactions less likely. RA factor and ANA were negative which made autoimmune diseases like SLE and RA less probable.

HLH is systemic immune hyperactivation syndrome, primary HLH occurs in individuals with genetic predisposition, viral infection, or malignancy, and secondary variant (MAS) is triggered by rheumatological conditions. As the deficient cytotoxic function leads to the deficiency in those cells to provide complete pathogen destruction alongside persistent lymphocyte and macrophage activation, those persistently activated macrophages cause tissue infiltration, production of ferritin, and high levels of TNF alpha and IL −6/18/8 in MAS. This broadly excessive cytokine production constitutes the root process for both AOSD and MAS. As per a retrospective cohort study of 206 patients with AOSD, including 20 who developed MAS, a ferritin threshold of 3500 mg/L had a sensitivity of 85% and a negative predictive value of 97% for identifying MAS [4]. ESR level was low in our patient which is the case for both AOSD and MAS, which is due to low or normal levels of haptoglobin and fibrinogen. Raised serum triglyceride level is considered to be a good marker of MAS, but it was not specifically analyzed in a flare of AOSD [5]. Meeting the criteria for AOSD with the markedly increased ferritin (38,291 ng/mL), makes our patient a case of MAS in AOSD.

Patients with moderate to severe AOSD manifest systemic signs like polyarthritis, persistent fever, and serositis. Anakinra, an IL‐1 receptor antagonist, is recommended, showing better remission induction than DMARDs alone, with alternatives Canakinumab, Rilonacept, and Tocilizumab. In MAS cases, which happens in 15% of AOSD patients, treatment differs. Current practice involves immunomodulators to control inflammation. High‐dose IV glucocorticoids remain the main MAS treatment, as in our patient. MAS as the first presentation of newly diagnosed AOSD is uncommon. In Nepal, considering rashes, fever, and joint pain, it is crucial to consider MAS in AOSD patients. Corticosteroid, often as sole treatment, shows a dramatic response [5, 6]. IVIG is effective when initiated early for both MAS and AOSD [7].

## 6 Conclusion

MAS is a life‐threatening and probably underdiagnosed complication of AOSD. In a patient with suspected AOSD, the occurrence during a disease flare of leukopenia or thrombocytopenia and extremely high serum ferritin levels should suggest MAS. The presence of hemophagocytosis on bone marrow biopsy is not mandatory for the diagnosis of MAS or HLH, as its sensitivity is low. Potential precipitating drugs should be withdrawn. First‐line treatment includes high‐dose steroids.

With studies showing a mortality rate of more than 50% in patients of AOSD with MAS which is five times more than the mortality rate with AOSD alone [8], understanding this combined picture and timely aggressive treatment is of utmost importance.

## Author Contributions

**Kanchan Bishwakarma:** conceptualization, data curation, methodology, resources, visualization, writing – original draft, writing – review and editing. **Kajal Bishwakarma:** conceptualization, data curation, investigation, resources, visualization, writing – original draft. **Sunil Bogati:** conceptualization, data curation, methodology, supervision, visualization, writing – original draft, writing – review and editing. **Saket Jha:** validation, visualization.

## Consent

Written informed consent was obtained from the patient to publish this report by the journal's patient consent policy.

## Conflicts of Interest

The authors declare no conflicts of interest.

## Data Availability

The authors have nothing to report.
